# Hybrid Molecular Mechanics/Coarse-Grained Simulations for Structural Prediction of G-Protein Coupled Receptor/Ligand Complexes

**DOI:** 10.1371/journal.pone.0047332

**Published:** 2012-10-19

**Authors:** Michael Leguèbe, Chuong Nguyen, Luciana Capece, Zung Hoang, Alejandro Giorgetti, Paolo Carloni

**Affiliations:** 1 INRIA Bordeaux Sud-Ouest, Institut de Mathématiques de Bordeaux, Talence, France; 2 Computational Biophysics, German Research School for Simulation Sciences, Jülich, Germany; 3 JARA - High-Performance Computing, Jülich, Germany; 4 International Centre for Genetic Engineering and Biotechnology, Trieste, Italy; 5 Vietnam National University Ho Chi Minh City, Ho Chi Minh City, Vietnam; 6 Department of Biotechnology, University of Verona, Verona, Italy; 7 Institute for Advanced Simulation, IAS-5 Intitute for Computational Biomedicine, Forschungszentrum Jülich, Jülich, Germany; German Cancer Research Center, Germany

## Abstract

Understanding how ligands bind to G-protein coupled receptors (GPCRs) provides insights into a myriad of cell processes and is crucial for drug development. Here we extend a hybrid molecular mechanics/coarse-grained (MM/CG) approach applied previously to enzymes to GPCR/ligand complexes. The accuracy of this method for structural predictions is established by comparison with recent atomistic molecular dynamics simulations on the human β2 adrenergic receptor, a member of the GPCRs superfamily. The results obtained with the MM/CG methodology show a good agreement with previous all-atom classical dynamics simulations, in particular in the structural description of the ligand binding site. This approach could be used for high-throughput predictions of ligand poses in a variety of GPCRs.

## Introduction

G-protein coupled receptors (GPCRs) are involved in an enormous number of biochemical processes at the cell membrane. They comprise the largest membrane protein superfamily across eukaryotes [1]. Human GPCRs are among the most important targets of pharmaceutical intervention, constituting the target for ∼30% of clinically used drugs [2]. Thus, methods for investigating how ligands bind to GPCRs are crucial not only for characterizing processes in cells but also for drug development.

Experimental structures of GPCRs are available for eleven members of that superfamily in eukaryotes (Table S1). Molecular dynamics (MD) simulations, based on structures predicted by bioinformatics methods, are often used to identify ligand poses on GPCRs for which experimental structural information is not available [3]. This approach can be very CPU intensive [4], especially to characterize large numbers of ligand/receptor complexes. On the other hand, coarse-grained (CG) approaches allow the study of large systems on longer timescales than those usually explored with MD [5], [6]. Indeed, the reduction of the number of degrees of freedom allows a reduction of the simulation time by ∼2 to ∼3 orders of magnitude compared to full atom force fields [7]. However, these approaches cannot describe the intermolecular ligand/protein interactions at atomic detail as required in ligand pose predictions.

**Figure 1:**
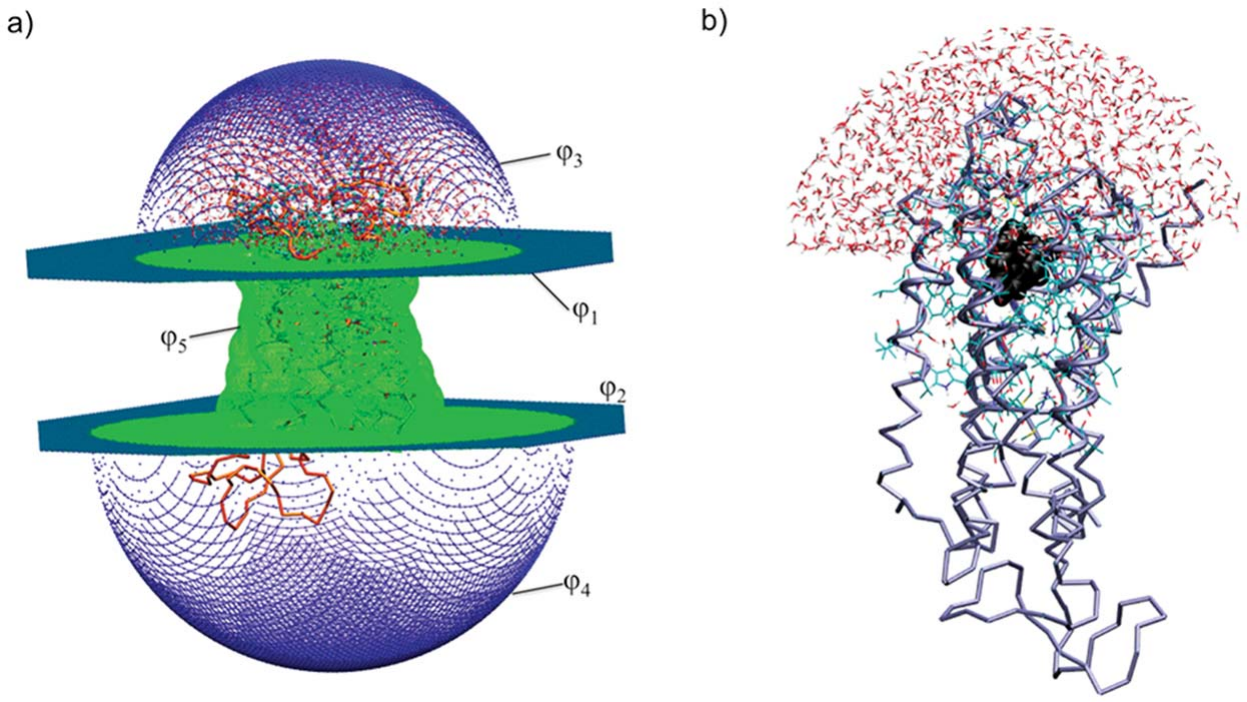
Simulated system. a) Five walls around the GPCR are used to mimic the presence of the lipid bilayer: the planar walls (*φ_1,2_*) are the dark green sheets located at the height of the membrane lipids head, the outer walls (*φ_3,4_*) are the dark blue hemispheres, and the membrane wall (*φ_5_*) is the surface in green. b) Model of hβ2-AR in complex with S-Carazolol (S-Car) for the MM/CG simulations; the protein backbone is represented by tubes, the MM and I regions and the water molecules are represented in licorice form, and the ligand S-Car is represented with black spheres.

A possible strategy to address this issue is to combine atomistic with CG modeling [8]–[14]. Molecular Mechanics/Coarse-Grained (MM/CG) simulation is an approach in which different representations of the system are modeled concurrently (Figure 1). A coupling scheme is used to connect the boundary of models. This approach has been developed for proteins by several groups, including ours [10]–[14]. In our scheme [12], [13], a region of interest (i.e. the active site of an enzyme, MM region) is treated at a molecular level using an atomistic force field. The one used here is the GROMOS96 43a1 force field [15], [16]. Hydration is accounted for including a droplet of water molecules around the MM region. The protein frame is described at a CG level using a Go-like model [17]. Such a model includes only the backbone *Cα* atoms. An interface (I region) is defined between the MM and CG regions to bridge the two different resolution models.

Our MM/CG approach was originally developed for enzymes where the MM region is exposed to the water solvent [13]. In GPCRs, the binding sites are located in the transmembrane region [18]. Thus, the presence of the lipid bilayer should be taken into account. In addition, the model requires some modifications to avoid the diffusion of water into the hydrophobic regions of the lipid bilayer. Hence, we have modified our MM/CG scheme and tested the accuracy of this method by comparing it with recent extensive atomistic molecular dynamics simulations [19] on the human β2 adrenergic receptor (hβ2-AR), a member of the GPCRs superfamily involved in cardiac functions [20], [21].

## Methods

### The MM/CG Scheme

The total potential energy of the system in the MM/CG approach is split into terms corresponding to different sets of atoms, belonging to the MM, CG and CG/I regions (see [12]) as follows (Eq. 1):
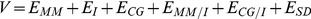
(1)


Here, the first term denotes the GROMOS96 force field [15]. Atoms in the interface region are represented at the atomistic level, thus the terms *E_I_* and *E_MM/I_* have the same form as *E_MM_*. *E_CG_* is given by a Go-like model [22] (Eq. 2):
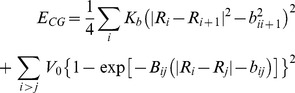
(2)


(i) The first term describes the interaction between consecutive CG beads (the C*α* atoms); *K_b_* is the force constant; *b_ij_* is the equilibrium distance, corresponding to the native distance between CG atoms; and (ii) Non-bonded interactions are taken into account in the second (Morse-potential-type) term: *V*_0_ = 5.3 kJ mol^−1^ is the well depth, and *B_ij_* is its modulating coefficient which is a function of *b_ij_*: *B_ij_* = 6/*b_ij_* (nm^−1^). These two parameters have been already used for both soluble and membrane enzymes [12], [13]. Here we consider the same value for *V*_0_ while *B_ij_* is set to 5+6/*b_ij_* (nm^−1^). This setup ensures the stability of the protein inside its transmembrane site. The interaction between the CG and I regions, described by the term *E_CG/I_*, is treated in the same form as *E_CG_*. The bonded terms are included between CG atoms and consecutive C*α* atoms of I region, and the non-bonded interactions between CG atoms and both C*α* and Cβ atoms [12]. This interface potential ensures the integrity of the backbone. The *E_SD_* term mimics the thermal and viscous solvent effects acting on the system [23].

To take into account the presence of the lipid bilayer, we have modified the potential energy function as follows.

1. We introduced five walls around the protein (Figure 1a). These are described by five functions *φ_i_*, (i = 1–5) using a level-set approach [24]. The region where the set of *φ_i_* is positive characterizes the protein site. The wall itself is the set of points for which the set *φ_i_* vanishes (Figure S1b). Two planar walls (*φ_i_*, i = 1,2) coincide with the height of the membrane lipid’s head. Two hemispheric walls ('outer walls’, *φ_i_*, i = 3,4) capping the extracellular and cytoplasmic ends of the protein (Figure 1a) are defined as follow:
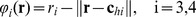
(3)where their locations are defined only outside the membrane region. The center chi of each hemisphere is located at the height of the membrane lipid heads, above/under the center of mass of the protein. The radius ri of each hemisphere is such that the minimum distance between any protein atoms and the wall is 20 Å. This creates a droplet of waters around the MM region similar to ref. [13]. The last wall (‘membrane wall’, φ5) follows the initial shape of the interface between protein and membrane (Figure 1a). This wall is defined as:
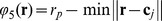
(4)where the distance between the point r and the closest initial position of Cα atoms cj is computed, and rp is a distance parameter that we have set to 2.0 Å. Additionally a smoothing technique (see Text S1 for further details) is applied to avoid the discontinuities in the wall.
2. Boundary potentials are added to the MM/CG potential energy function. They are defined as functions of the distance (*V_i_*(*d*)(i = 1–5), *d* = min(*φ_i_*, i = 1–5) from the corresponding walls, as follows: (Figure S1)

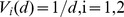
(5)

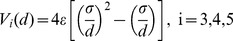
(6)


The index *i* of boundary potential corresponds to the index of the surfaces *φ_i_*. The potential applied to an atom is the one corresponding to the closest wall from that atom *d = *min *φ_i_* (i = 1,5). *V_i_* (i = 1,2) is purely repulsive; *V_i_* (i = 3,4,5) is a softened Lennard-Jones-type potential; *ε* is the depth of the potential well; and *σ* is the finite distance at which the potential *V_i_* (i = 3,4,5) is zero. The minimum of the potential is at *d = r_p_*. Waters, *α*-carbons of both MM and CG regions, and atoms belonging to external aromatic residues TRP and TYR are influenced by these potentials. The potential on the membrane wall is represented by a softened Lennard-Jones potential, *V_5_*. It constrains the shape of the protein while providing a good degree of flexibility. This model neither includes electrostatics nor allows distinguishing between different types of bilayers.

The force due to the presence of the wall is derived from the potentials *V_i_*:
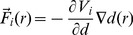
(7)


The cutoff distance of the force is set to 7 Å for the repelling walls *V_i_* (i = 1,2), and to 1.5*r_p_* for the outer walls and membrane wall *V_i_* (i = 3,4,5). The first value is chosen such that a water molecule can not pass through this distance during one time step, while the second value guarantees that the force does not affect the MM region. The force is shifted so that it is continuous at the cutoff distance, to avoid a sharp disruption. In addition, it is set to a finite value (1000 kJ mol^−1^ nm^−1^) near the wall to prevent too large of a force being applied to the system.

**Figure 2:**
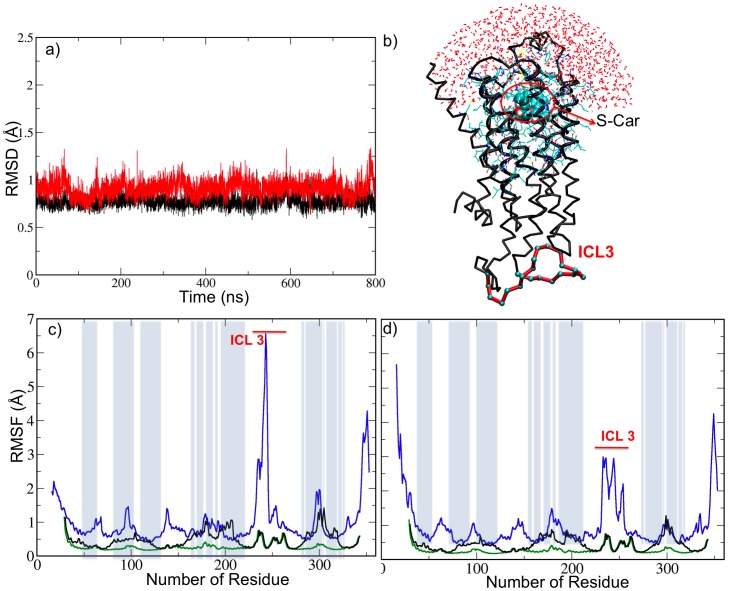
Results of the simulations. a) Root-mean-square-deviation of hβ2-AR’s backbone atoms in the MM/CG simulation of hβ2-AR.S-Car (black lines) and hβ2-AR.R-ISO (red lines), relative to the initial X-ray structure, plotted as a function of time. b) MM/CG representation of the hβ2-AR.S-Car complex. The MM and I regions, together with the water molecules, are shown in a line representation, the ligand S-Car is shown as spheres, and the ICL3 is highlighted in red. c) and d) Root Mean-Square Fluctuations of hβ2-AR’s backbone atoms calculated based on MD simulations. Results for all-atom simulations [19], MM/CG simulations and CG simulations are shown in blue, black and green lines respectively. Results for hβ2-AR.R-ISO and hβ2-AR.S-Car complexes are shown in panels c) and d), respectively. Residues included in the MM and I regions (which feature all-atom representation) are highlighted by grey bars on the plots.

### MM/CG Simulations of Human β2-adrenergic Receptor (hβ2AR) in Complex with S-carazolol (S-Car) or R-isoprenaline (R-Iso)

The calculations are based on the X-ray structure of the hβ2AR in complex with S-Car (PDB code 2RH1) [25]. Since the third intracellular loop (ICL3, residues 231–262) is not present in the structure, it was predicted using the Modeller9v3 program [26]. The structure of the complex between the hβ2AR and the agonist R-Iso was obtained following the procedure of [19]. 975 SPC water molecules [27] were added. This constitutes a layer of approximately ∼15 Å around the MM region. Similarly to our previous works [12], [13], the MM region included all the residues at a distance less than 5 Å from the bound ligand, which consisted in residues 79–82, 86, 109 to 118, 164–165, 193–195, 199–208, 282, 286, 289–290, 293, 308, 311–316, the corresponding ligands R-Iso or S-Car, and the water molecules. A cutoff of 6 Å (as measured from the MM boundary) was applied in order to calculate the residues included in the interface between the MM and the CG subsystems. The MM and I regions were treated using the GROMOS96 43a1 force field [15], [16]. Our MM/CG scheme has been extensively tested for this force field [12], [13]. Structural properties for a large number of proteins do not change by a large amount when using updated versions of this force field, such as 53a5 and 53a6 [16]. The resulting total number of atoms is 4597 and 4587 for the hβ2AR.S-Car and hβ2AR.R-ISO complexes respectively. Starting from this structure, 800 ns MM/CG simulations were performed using a 2 fs time step. The protein complexes were encapsulated in a ∼31 Å thick implicit membrane, with the transmembrane wall 2.0 Å from the C*α* atoms. Cutoffs of 16 Å were used for the electrostatic, van der Waals and Go-like interactions. The SHAKE algorithm [28] was used to fix the distance in bonds containing hydrogen(s). The temperature was set to 300 K using stochastic dynamics, controlled by an inverse friction constant with a value of 0.4 ps. Periodic boundary conditions were used. RESP charges [29], [30] for the ligands were taken from ref. [19]. All simulations have been performed using our MM/CG implementation in Gromacs 4.5.1 [31].

CG simulations were carried out for up to 1 µs using a 2 fs time-step. Within this Go-model, each residue is treated as one bead at the position of the C*α* atom. Therefore the atom number of this system is 314, which is the number of residues present in X-ray structure.

**Figure 3:**
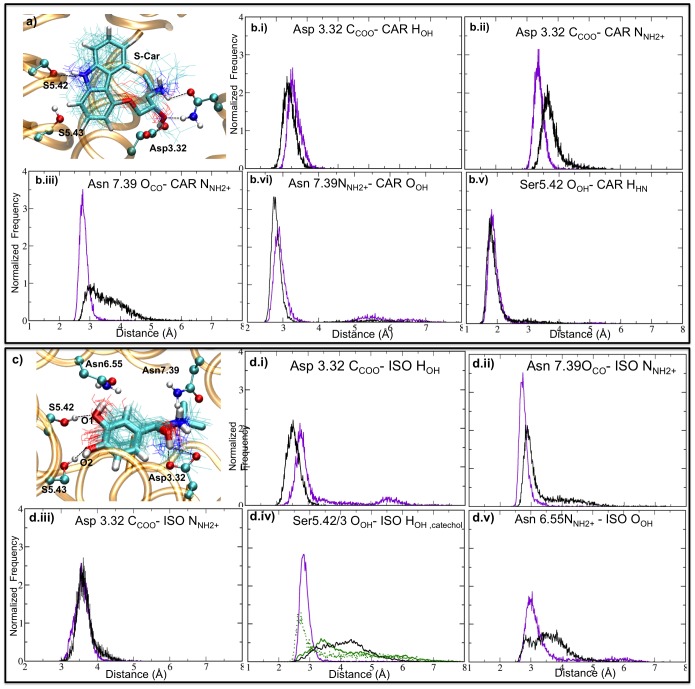
MM/CG and MD simulations of hβ2-AR.S-Car and hβ2-AR.R-Iso complexes. H-bond interactions between S-Car and hβ2-AR, reported in ref. [19]. Panels a) and c) display snapshots of the binding site, obtained from the MM/CG trajectory of the S-Car and R-Iso complexes respectively. Superimposed positions of the agonist and reverse agonist along the trajectories are shown in lines representation (snapshots were taken every 40 ns). The distribution of all H-bonds and salt involved in S-Car and R-Iso binding are shown in Panels b.i) to b.v) and d.i) to d.v). Results of the MM/CG and the all-atom MD simulations [19] are shown in black and violet lines respectively. In d.iv), black and violet lines correspond to the distance between the O1 (labeled in panel a) and the OH group of Ser5.42 in the MM/CG simulations, and the O2 and the OH group of Ser5.42 in the all-atom simulations, respectively. The distance between O1 and Ser5.43-OH and between O2 and Ser5.43-OH in the MM/CG simulation are shown in full and dotted green lines respectively.

## Results and Discussion

The accuracy of this method was established by performing MM/CG simulations of the human GPCR β2 adrenergic receptor (hβ2-AR) in complex with its inverse agonist S-Carazolol (S-Car) and its agonist R-Isoprenaline (R-ISO), and by comparing these with a 800 ns all atom simulation of this system embedded in a lipid bilayer [19]. Both the all-atom MD and the MM/CG calculations are based on the hβ2-AR/S-Car complex X-ray structure [25].

The MM/CG simulations were carried out for up to 800 ns. The MM region consisted of 476 and 486 atoms, while the overall system was made of 4597 and 4587 atoms for the hβ2-AR.S-Car and hβ2-AR.R-Iso complexes respectively. This allows us to run more than 80 ns/day on 16 CPUs (2.93 GHz Intel Xeon), a 15-fold speedup compared to MD simulations of the same system (Ursula Roethlisberger, private communication). With respect to this speed-up, we notice that a significant speed-up could also have been obtained using an all-atom representation for the whole protein while adding waters on both the intracellular and the extracellular region. However, the combined MM/CG representation allows a further reduction of the system size, since (i) waters are only needed in the extracellular site of the system; and (ii) the use of the CG representation for residues far away from the binding cavity reduces the system size.

Along the MM/CG trajectories, both complex structures remain stable, as observed in the root-mean-square deviation calculation (Figure 2a and Figure S4). The residue root-mean-square fluctuation calculated using the MM/CG approach follows the trend obtained with all-atom MD (Figure 2c,d). The results in panels c) and d) show that globally the fluctuations of the MM and I regions are similar to the fluctuations observed in the all-atom MD simulations. In the CG regions, the fluctuations are much lower, possibly due to the higher rigidity of the CG Go-model force field in comparison to the all-atom force field [12]. This is observed in the full CG simulations (green line in Figure 2c,d). The main discrepancies between all atom MD and MM/CG simulations are observed in the intracellular loop 3 (ICL3, ranging from residues 233 to 253 approximately), which exhibits larger flexibility in the all-atom simulations. As shown in panel b), the ICL3, which is not present in the crystal structure [25], is located in the cytoplasmic region far away from the binding cavity. Thus, it was included in the CG region, and it is not expected to directly affect the properties of the binding site. Small differences in the fluctuations (smaller than 1Å) observed in the MM region with respect to the all-atom simulation can be expected due to the different force fields applied in both simulations (Amber99 [32] and Gromos43a1 [15], respectively). The differences observed here are of the same order of magnitude as those observed in other comparisons between different force fields [33]. Indeed, these differences were also observed in two other independent test simulations of the hβ2-AR/S-Car complex (Figure S5). The velocity autocorrelation function and the radial distribution function for the water molecules (Figure S2, Table S2) do not differ significantly from the data obtained for bulk water [34], [35]. This suggests that the MM/CG scheme does not significantly constraint either the structure or the mobility of water molecules.

The structural determinants at the active site were well maintained (Figure 3). The interactions between the ligands and the protein matrix evolved in good agreement with the all-atom simulations. These included H-bond interactions between the ligands and residues Asn7.39, Asp3.32, Ser5.42, Ser5.43 and Asn6.55 (the last two residues, only in the case of the hβ2-AR.R-Iso complex). However, small differences have been observed in some specific interactions. The H-bonds between Asn7.39 carbonyl group (O_CO_) and the NH_2_^+^ group of the agonist (R-Iso) or the reverse agonist (S-car) are only partially formed in the MM/CG simulation. (Figure 3 panel b.iii, d.ii). The H-bond between NH_2_^+^ group of Asn6.55 and R-Iso.OH is longer in the MM/CG simulations than in the all-atom simulations (Figure 3, panel d.v). Finally, both R-Iso.OH groups form H-bonds to Ser5.42 and Ser5.43 in the MM/CG simulation, while only the R-Iso.O(2)H group forms an H-bond to Ser5.42 in the all-atom simulation (Figure 3, panel d.iv) [19]. The fact that the simulations performed in this work show a high level of agreement with the all-atom simulations allows us to suggest that the results do not critically depend on the choice of the force field.

To investigate the predictive power of the method at the structural level, we ran an additional simulation in which we located the ligand S-Car in a position different from the crystallographic pose (Figure S3). In this new position, none of the interactions with the residues found in the X-ray structure of hβ2-AR/S-Car complex [25] are present. In these new simulations, the ligand migrates to the correct pose between 150 and 200 ns, forming the key interactions with Ser5.42, Ser5.43, Asp 3.32 and Asn7.39 (Figure S3). Hence, our method is not only able to conserve the ligand-receptor structure but also able to predict the correct pose of the ligand in the binding site.

All together, the MM/CG simulations reproduce the key structural features of the active site found in the MD simulations. The introduction of the potential wall to represent the membrane leads to a large reduction in the computational cost of the simulation, conserving the stability of the protein structure. Moreover, the ligand remains in a stable position inside the binding cavity throughout the long-scale simulation, conserving the key interactions with the protein matrix at the binding site. Due to its low cost and high accuracy, this method can be applied in this context to the study of a large number of GPCRs-ligand complexes.

### Conclusions

We have presented a hybrid MM/CG method to investigate hβ2-AR, a receptor from the GPCR superfamily. The method allows a large speed up of the simulation while conserving all the key information related to the ligand-receptor interaction. This method can be extended to a large number of GPCR/ligand complexes and may be very useful in computer-aided drug design. Our code is implemented in GROMACS 4.5 [31] and is freely available upon request.

Combining MM/CG with model-built structures from homology modeling and/or molecular docking (such as in ref. [36]) may be a useful tool for structural predictions of GPCR/ligand complexes. The method allows the efficient and relatively cheap sampling of side chains orientations at the binding site while fully including hydration. This is particularly important for GPCRs, as water molecules can be found at the binding site of these receptors, waters which may be crucial to stabilizing the ligand [37], [38].

## Supporting Information

Figure S1 Wall potentials. *(A)* Schematic of the wall potentials *V*_i_(*d*) (i = 1,2) plotted as a function of distance to the walls *d*. *(B)* The membrane wall *φ_5_* and the potential *V*_5_(*d*) shifted by 2*r_p_*.
(TIFF)

Figure S2 Velocity autocorrelation (C(t)) function for the oxygen atoms of the water molecules in the MM/CG simulation of the hB2-AR/S-Car complex. The correlation of the velocities is lost after ∼0.6 ps, in agreement with the results previously obtained for a solution of SPC waters [35].
(TIF)

Figure S3 MM/CG simulation of hB2-AR/S-Car complex. Here the S-Car ligand is originally located at a position different from the crystallographic pose. Panels a and b show snapshots taken at 0 ns and 180 ns of the simulation. Panel c shows the RMSD of the S-Car ligand with respect to the crystallographic position.
(TIF)

Figure S4 Root-mean-square-deviation per residue of hβ2-AR’s backbone atoms in the MM/CG simulation of a) hβ2-AR.S-Car and b) hβ2-AR.R-ISO relative to the initial X-ray structure. Overall, the protein’s residues remain close to the crystal structure, with an RMSD lower than ∼2 Å. The regions with higher fluctuations with respect to the crystal structure consist of residues ∼158 to 200 (in helix IV), and ∼300–305 (N-terminal extreme of helix VII), in agreement with the results presented in Figure 2c,d. These regions also show fluctuations in the all-atom simulations (as observed in Figures 2c,d), and do not include any of the residues interacting directly with the ligand in the binding cavity.
(TIF)

Figure S5 Root-mean-square fluctuation (RMSF) of the backbone atoms calculated for two independent 400 and 800 ns simulations of the hB2-AR/S-Car complex, shown with cyan and red lines respectively. For comparison purposes, the RMSF calculated for the all-atom simulation and for the MM/CG simulation described in the main text are shown with blue and black lines respectively. Grey bars indicate the MM and I regions. No large differences among the MM/CG simulations are observed.
(TIF)

Table S1 Available GPCR's structures in Protein Data Bank (PDB), adapted from http://blanco.biomol.uci.edu/mpstruc on 14 April 2012.
(DOC)

Table S2 Maximum and minimum positions (in Å) of the Oxygen-Oxygen radial distribution functions for the oxygen atoms of the water molecules in the MM/CG simulation of the hB2-AR/S-Car complex. The values of the positions obtained for a solution of SPC waters are shown for comparison, which are taken from reference [34].
(DOC)

Methods S1 Details on the MM/CG parameters (DOC).
(DOC)
