# Supplementary figures and images for: Hybrid Molecular Mechanics/Coarse-Grained Simulations for Structural Prediction of G-Protein Coupled Receptor/Ligand Complexes
Source: PLoS One. 2012 Oct 19;7(10):e47332. doi: 10.1371/journal.pone.0047332 (PMC3477165; doi:10.1371/journal.pone.0047332)

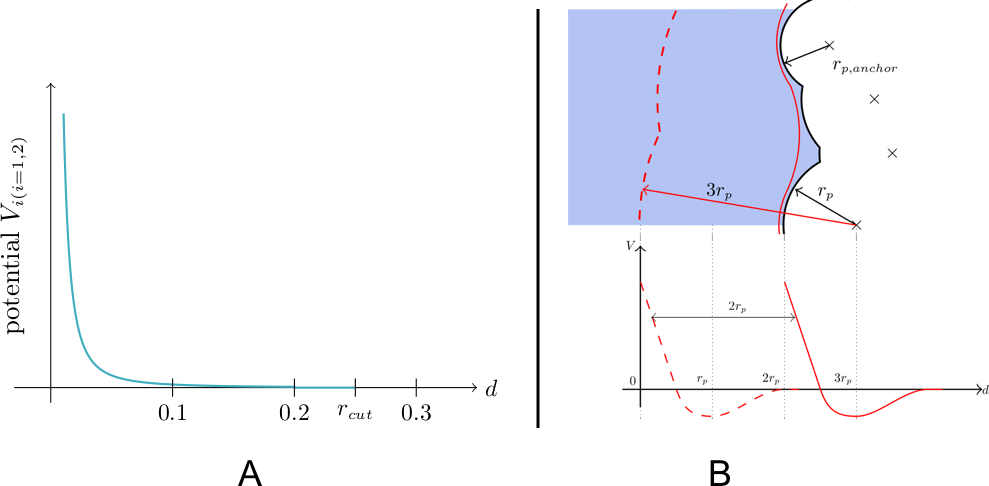

Supplement: Figure S1 — Wall potentials. (A) Schematic of the wall potentials V i(d) (i = 1,2) plotted as a function of distance to the walls d. (B) The membrane wall φ5 and the potential V 5(d) shifted by 2rp. (TIFF) [file pone.0047332.s001.tif]

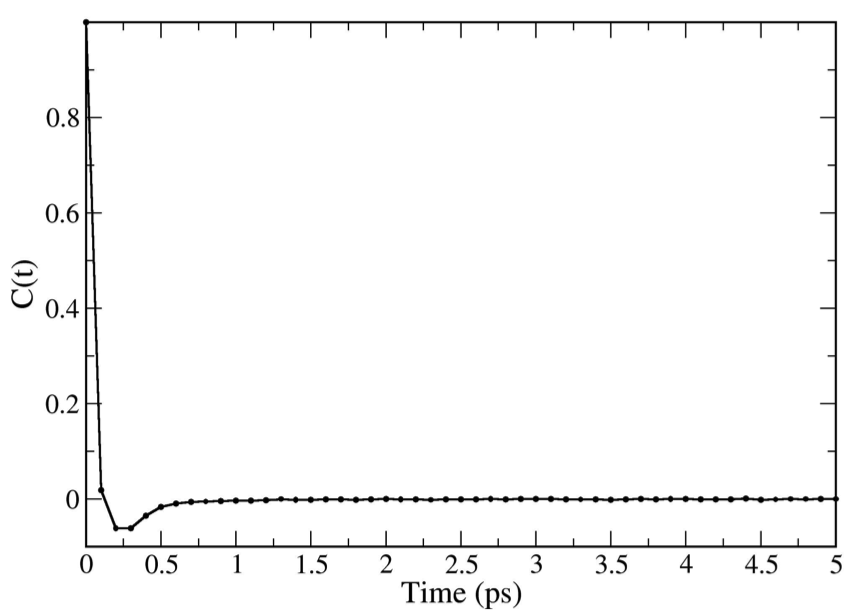

Supplement: Figure S2 — Velocity autocorrelation (C(t)) function for the oxygen atoms of the water molecules in the MM/CG simulation of the hB2-AR/S-Car complex. The correlation of the velocities is lost after ∼0.6 ps, in agreement with the results previously obtained for a solution of SPC waters [35]. (TIF) [file pone.0047332.s002.tif]

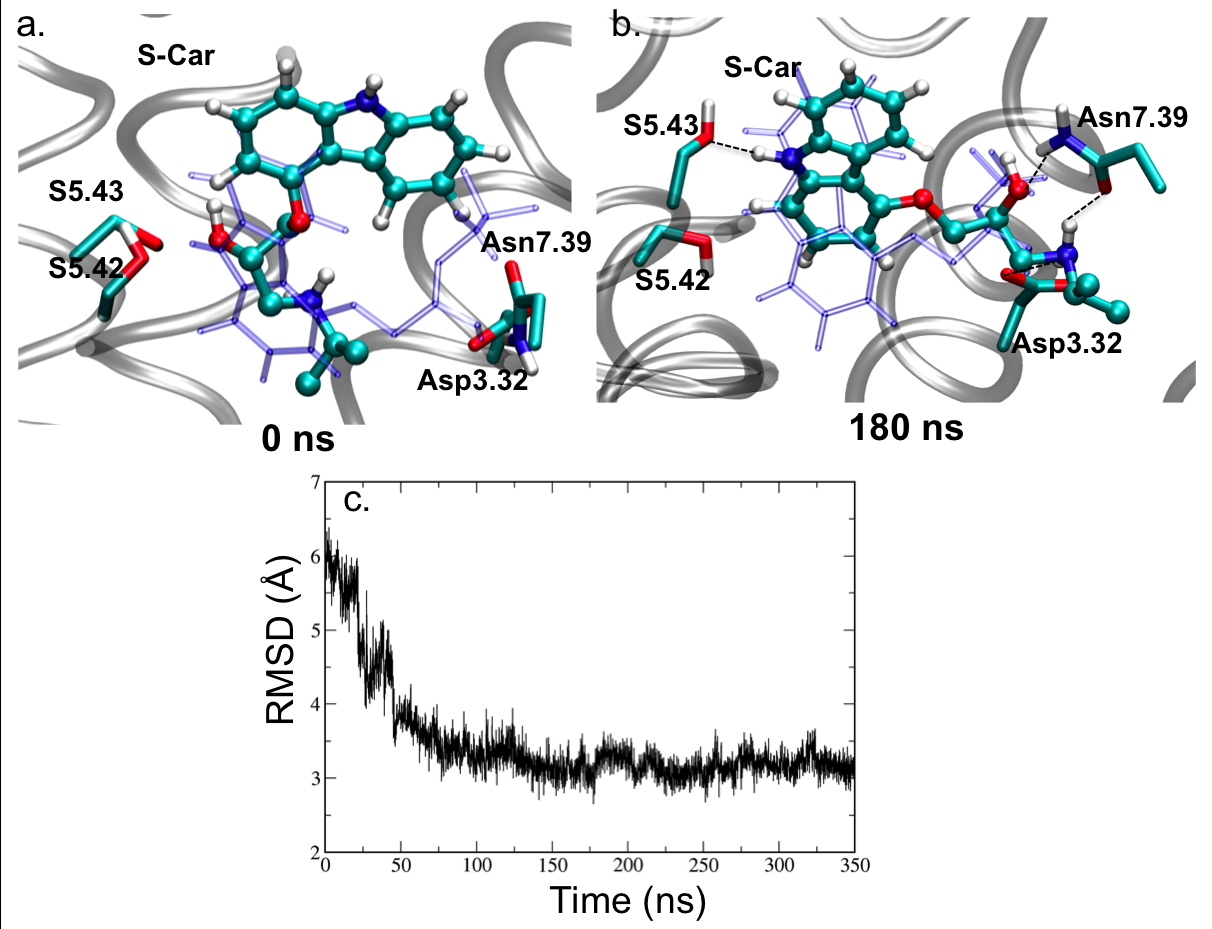

Supplement: Figure S3 — MM/CG simulation of hB2-AR/S-Car complex. Here the S-Car ligand is originally located at a position different from the crystallographic pose. Panels a and b show snapshots taken at 0 ns and 180 ns of the simulation. Panel c shows the RMSD of the S-Car ligand with respect to the crystallographic position. (TIF) [file pone.0047332.s003.tif]

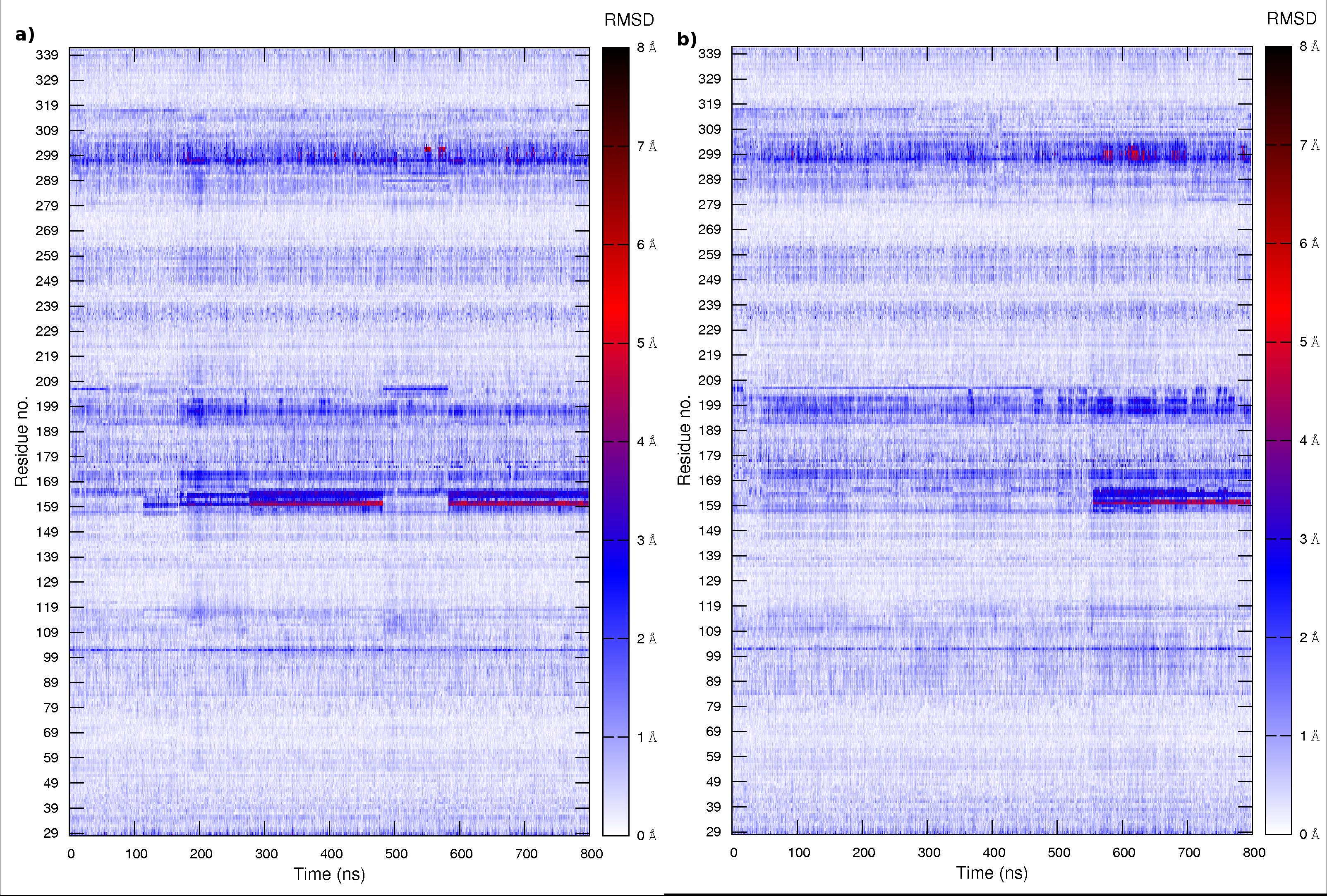

Supplement: Figure S4 — Root-mean-square-deviation per residue of hβ2-AR’s backbone atoms in the MM/CG simulation of a) hβ2-AR.S-Car and b) hβ2-AR.R-ISO relative to the initial X-ray structure. Overall, the protein’s residues remain close to the crystal structure, with an RMSD lower than ∼2 Å. The regions with higher fluctuations with respect to the crystal structure consist of residues ∼158 to 200 (in helix IV), and ∼300–305 (N-terminal extreme of helix VII), in agreement with the results presented in Figure 2c,d. These regions also show fluctuations in the all-atom simulations (as observed in Figures 2c,d), and do not include any of the residues interacting directly with the ligand in the binding cavity. (TIF) [file pone.0047332.s004.tif]

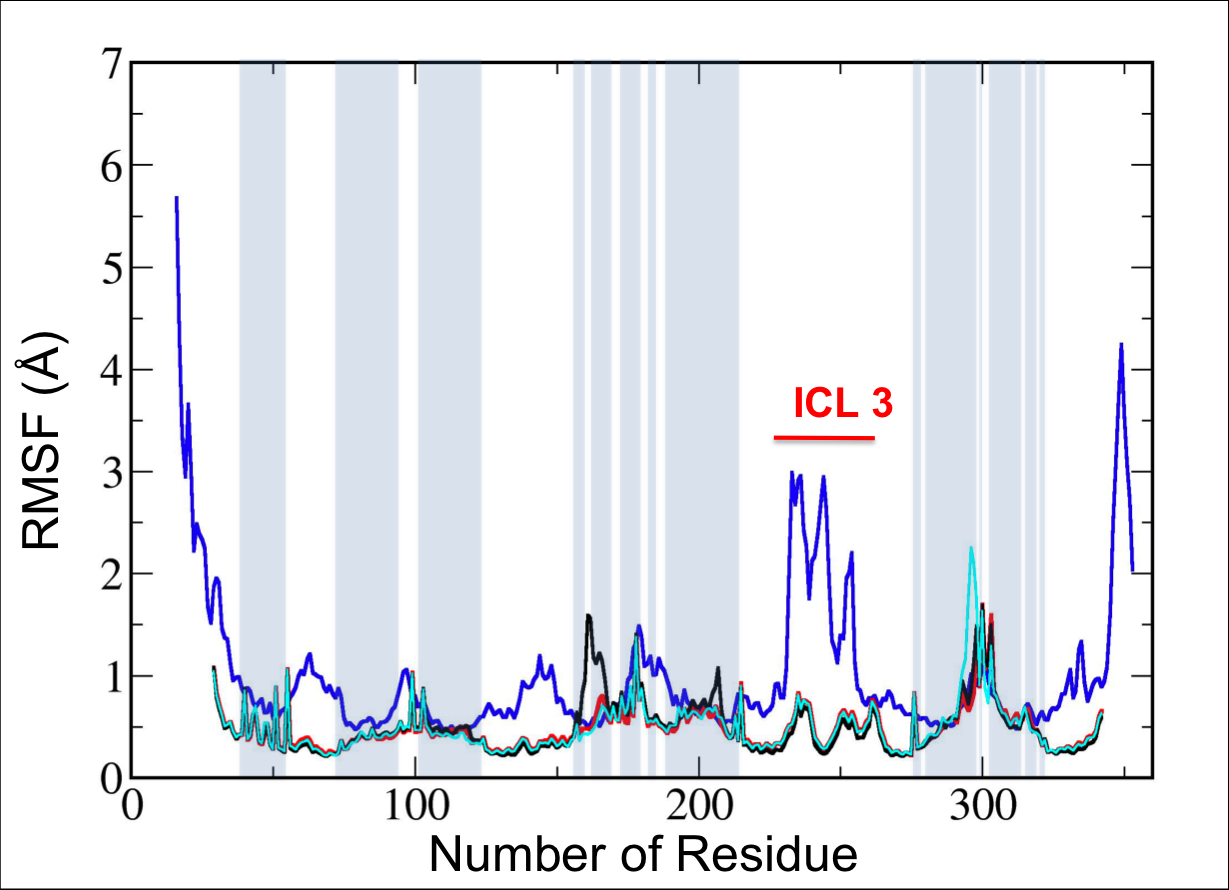

Supplement: Figure S5 — Root-mean-square fluctuation (RMSF) of the backbone atoms calculated for two independent 400 and 800 ns simulations of the hB2-AR/S-Car complex, shown with cyan and red lines respectively. For comparison purposes, the RMSF calculated for the all-atom simulation and for the MM/CG simulation described in the main text are shown with blue and black lines respectively. Grey bars indicate the MM and I regions. No large differences among the MM/CG simulations are observed. (TIF) [file pone.0047332.s005.tif]
