# Supplementary material for: Hybrid Molecular Mechanics/Coarse-Grained Simulations for Structural Prediction of G-Protein Coupled Receptor/Ligand Complexes
Source: PLoS One. 2012 Oct 19;7(10):e47332. doi: 10.1371/journal.pone.0047332 (PMC3477165; doi:10.1371/journal.pone.0047332)
Supplement: Table S1 — Available GPCR's structures in Protein Data Bank (PDB), adapted from http://blanco.biomol.uci.edu/mpstruc on 14 April 2012. (DOC) [file pone.0047332.s006.doc]

| Protein name | Organism | PDB code | Resolution (Å) |
| --- | --- | --- | --- |
| Rhodopsin | *Bovine (Bos taurus)* | 1F88 [1]  1L9H [2]  1GZM [3]  1U19 [4]  2J4Y [5]  2I37 [6]  3CAP [7]  3PXO [8]  3DQB [9] | 2.80  2.60  2.65  2.20  3.40  4.15  2.90  3.00  3.20 |
| Rhodopsin | *Squid (Todarodes pacificus)* | 2Z73 [10]  2ZIY [11] | 2.50  3.70 |
| β_1_ adrenergic receptor | *Meleagris gallopavo (turkey)* | 2VT4 [12]  2Y00 [13]  2YCW [14] | 2.70  2.50  3.00 |
| β_2_ adrenergic receptor | *Homo sapiens* | 2R4R [15]  3KJ6 [16]  2RH1 [17]  3D4S [18]  3P0G [19]  3PDS [20] 3SN6 [21] | 3.40  3.40  2.40  2.80  3.50  3.50  3.20 |
| A_2A_ adenosine receptor | *Homo sapiens* | 3EML [22] 3QAK [23] 2YDO [24] 3RFM [25] | 2.60  2.71  3.00  3.60 |
| CXCR4 Chemokine Receptor | *Homo sapiens* | 3ODU [26] | 2.50 |
| Dopamine D3 Receptor | *Homo sapiens* | 3PBL [27] | 2.89 |
| Histamine H_1_ receptor | *Homo sapiens* | 3RZE [28] | 3.10 |
| M2 muscarinic acetylcholine receptor | *Homo sapiens* | 3UON [29] | 3.00 |
| κ-opioid receptor | *Homo sapiens* | 4DJH [30] | 2.90 |
| μ-opioid receptor | *Mus musculus* | 4DKL [31] | 2.80 |

**Table S1.** Available GPCR's structures in Protein Data Bank (PDB), adapted from http://blanco.biomol.uci.edu/mpstruc on 14 April 2012.

**References:**

1. Palczewski K, Kumasaka T, Hori T, Behnke CA, Motoshima H, et al. (2000) Crystal structure of rhodopsin: A G protein-coupled receptor. Science 289: 739–745.

2. Okada T, Fujiyoshi Y, Silow M, Navarro J, Landau EM, et al. (2002) Functional role of internal water molecules in rhodopsin revealed by X-ray crystallography. Proc Natl Acad Sci USA 99: 5982–5987. doi:10.1073/pnas.082666399.

3. Li J, Edwards PC, Burghammer M, Villa C, Schertler GFX (2004) Structure of bovine rhodopsin in a trigonal crystal form. J Mol Biol 343: 1409–1438. doi:10.1016/j.jmb.2004.08.090.

4. Okada T, Sugihara M, Bondar A-N, Elstner M, Entel P, et al. (2004) The retinal conformation and its environment in rhodopsin in light of a new 2.2 A crystal structure. J Mol Biol 342: 571–583. doi:10.1016/j.jmb.2004.07.044.

5. Standfuss J, Xie G, Edwards P, Burghammer M, Oprian D, et al. (2007) Crystal structure of a thermally stable rhodopsin mutant. Journal of Molecular Biology 372: 0. doi:10.1016/j.jmb.2007.03.007.

6. Salom D, Lodowski DT, Stenkamp RE, Le Trong I, Golczak M, et al. (2006) Crystal structure of a photoactivated deprotonated intermediate of rhodopsin. Proc Natl Acad Sci USA 103: 16123–16128. doi:10.1073/pnas.0608022103.

7. Park JH, Scheerer P, Hofmann KP, Choe H-W, Ernst OP (2008) Crystal structure of the ligand-free G-protein-coupled receptor opsin. Nature 454: 183–187. doi:10.1038/nature07063.

8. Choe H-W, Kim YJ, Park JH, Morizumi T, Pai EF, et al. (2011) Crystal structure of metarhodopsin II. Nature 471: 651–655. doi:10.1038/nature09789.

9. Scheerer P, Park JH, Hildebrand PW, Kim YJ, Krauss N, et al. (2008) Crystal structure of opsin in its G-protein-interacting conformation. Nature 455: 497–502. doi:10.1038/nature07330.

10. Murakami M, Kouyama T (2008) Crystal structure of squid rhodopsin. Nature 453: 363–367. doi:10.1038/nature06925.

11. Shimamura T, Hiraki K, Takahashi N, Hori T, Ago H, et al. (2008) Crystal structure of squid rhodopsin with intracellularly extended cytoplasmic region. J Biol Chem 283: 17753–17756. doi:10.1074/jbc.C800040200.

12. Warne T, Serrano-Vega MJ, Baker JG, Moukhametzianov R, Edwards PC, et al. (2008) Structure of a beta1-adrenergic G-protein-coupled receptor. Nature 454: 486–491. doi:10.1038/nature07101.

13. Warne T, Moukhametzianov R, Baker JG, Nehmé R, Edwards PC, et al. (2011) The structural basis for agonist and partial agonist action on a β(1)-adrenergic receptor. Nature 469: 241–244. doi:10.1038/nature09746.

14. Moukhametzianov R, Warne T, Edwards PC, Serrano-Vega MJ, Leslie AGW, et al. (2011) Two distinct conformations of helix 6 observed in antagonist-bound structures of a beta1-adrenergic receptor. Proc Natl Acad Sci USA 108: 8228–8232. doi:10.1073/pnas.1100185108.

15. Rasmussen SGF, Choi H-J, Rosenbaum DM, Kobilka TS, Thian FS, et al. (2007) Crystal structure of the human beta2 adrenergic G-protein-coupled receptor. Nature 450: 383–387. doi:10.1038/nature06325.

16. Bokoch M, Zou Y, Rasmussen S, Liu C, Nygaard R, et al. (2010) Ligand-specific regulation of the extracellular surface of a G-protein-coupled receptor. Nature 463: 108–112.

17. Cherezov V, Rosenbaum DM, Hanson MA, Rasmussen SGF, Thian FS, et al. (2007) High-Resolution Crystal Structure of an Engineered Human 2-Adrenergic G Protein Coupled Receptor. Science 318: 1258–1265. doi:10.1126/science.1150577.

18. Hanson M, Cherezov V, Griffith M, Roth C, Jaakola V-P, et al. (2008) A specific cholesterol binding site is established by the 2.8 A structure of the human beta2-adrenergic receptor. Structure 16: 0. doi:10.1016/j.str.2008.05.001.

19. Rasmussen SGF, Choi H-J, Fung JJ, Pardon E, Casarosa P, et al. (2011) Structure of a nanobody-stabilized active state of the β(2) adrenoceptor. Nature 469: 175–180. doi:10.1038/nature09648.

20. Rosenbaum DM, Zhang C, Lyons JA, Holl R, Aragao D, et al. (2011) Structure and function of an irreversible agonist-β(2) adrenoceptor complex. Nature 469: 236–240. doi:10.1038/nature09665.

21. Rasmussen SGF, Devree BT, Zou Y, Kruse AC, Chung KY, et al. (2011) Crystal structure of the β(2) adrenergic receptor-Gs protein complex. Nature. doi:10.1038/nature10361.

22. Jaakola V-P, Griffith MT, Hanson MA, Cherezov V, Chien EYT, et al. (2008) The 2.6 angstrom crystal structure of a human A2A adenosine receptor bound to an antagonist. Science 322: 1211–1217. doi:10.1126/science.1164772.

23. Xu F, Wu H, Katritch V, Han GW, Jacobson KA, et al. (2011) Structure of an agonist-bound human A2A adenosine receptor. Science 332: 322–327. doi:10.1126/science.1202793.

24. Lebon G, Warne T, Edwards PC, Bennett K, Langmead CJ, et al. (2011) Agonist-bound adenosine A2A receptor structures reveal common features of GPCR activation. Nature 474: 521–525. doi:10.1038/nature10136.

25. Doré AS, Robertson N, Errey JC, Ng I, Hollenstein K, et al. (2011) Structure of the adenosine A(2A) receptor in complex with ZM241385 and the xanthines XAC and caffeine. Structure 19: 1283–1293. doi:10.1016/j.str.2011.06.014.

26. Wu B, Chien EYT, Mol CD, Fenalti G, Liu W, et al. (2010) Structures of the CXCR4 chemokine GPCR with small-molecule and cyclic peptide antagonists. Science 330: 1066–1071. doi:10.1126/science.1194396.

27. Chien EYT, Liu W, Zhao Q, Katritch V, Han GW, et al. (2010) Structure of the human dopamine D3 receptor in complex with a D2/D3 selective antagonist. Science 330: 1091–1095. doi:10.1126/science.1197410.

28. Shimamura T, Shiroishi M, Weyand S, Tsujimoto H, Winter G, et al. (2011) Structure of the human histamine H1 receptor complex with doxepin. Nature 475: 65–70. doi:10.1038/nature10236.

29. Haga K, Kruse AC, Asada H, Yurugi-Kobayashi T, Shiroishi M, et al. (2012) Structure of the human M2 muscarinic acetylcholine receptor bound to an antagonist. Nature. doi:10.1038/nature10753.

30. Wu H, Wacker D, Mileni M, Katritch V, Han GW, et al. (2012) Structure of the human κ-opioid receptor in complex with JDTic. Nature. doi:10.1038/nature10939.

31. Manglik A, Kruse AC, Kobilka TS, Thian FS, Mathiesen JM, et al. (2012) Crystal structure of the µ-opioid receptor bound to a morphinan antagonist. Nature: –. doi:10.1038/nature10954.
