# Supplementary material for: Hybrid Molecular Mechanics/Coarse-Grained Simulations for Structural Prediction of G-Protein Coupled Receptor/Ligand Complexes
Source: PLoS One. 2012 Oct 19;7(10):e47332. doi: 10.1371/journal.pone.0047332 (PMC3477165; doi:10.1371/journal.pone.0047332)
Supplement: Table S2 — Maximum and minimum positions (in Å) of the Oxygen-Oxygen radial distribution functions for the oxygen atoms of the water molecules in the MM/CG simulation of the hB2-AR/S-Car complex. The values of the positions obtained for a solution of SPC waters are shown for comparison, which are taken from reference [34]. (DOC) [file pone.0047332.s007.doc]

|  | MM/CG simulation | Solution |
| --- | --- | --- |
| First Maximum | 2.75 | 2.78 |
| First Minimum | 3.45 | 3.55 |
| Second Maximum | 4.45 | 4.50 |

**Table S2.** Maximum and minimum positions (in Å) of the Oxygen-Oxygen radial distribution functions for the oxygen atoms of the water molecules in the MM/CG simulation of the hB2-AR/S-Car complex. The values of the positions obtained for a solution of SPC waters are shown for comparison, which are taken from reference [5].
