# Supplementary material for: Hybrid Molecular Mechanics/Coarse-Grained Simulations for Structural Prediction of G-Protein Coupled Receptor/Ligand Complexes
Source: PLoS One. 2012 Oct 19;7(10):e47332. doi: 10.1371/journal.pone.0047332 (PMC3477165; doi:10.1371/journal.pone.0047332)
Supplement: Methods S1 — Details on the MM/CG parameters (DOC). (DOC) [file pone.0047332.s008.doc]

Supporting Information

*Details on the MM/CG parameters*

The MM/CG method has been successfully applied for soluble proteins [1] and membrane proteins [2] for which the binding site is located outside of the membrane region. All the parameters from this model are kept in our version except for the modulating coefficient in the Morse-potential-type *B_ij_* (see Eq. 2). By increasing the value of this parameter, the attraction among CG beads increases more rapidly when atoms move away from their equilibrium distances. We find that increasing the value of *B_ij_* from 6/*b_ij_* (nm^-1^) to 5 + 6/*b_ij_* (nm^-1^) ensures the stability of the protein inside the transmembrane site.

The main goal of our set-up is to prevent water molecules from diffusing (i) away from the protein and (ii) into the hydrophobic region of the membrane. Regarding the former issue, simple geometries of repulsive walls are sufficient to keep waters from diffusing away. These are the surfaces *φ_1_* to *φ_4_* in Figure 1. The surfaces *φ_1_* and *φ_2_* are defined by the two planes on each side of membrane. The surfaces *φ_3_ and φ_4_* are two hemispheres added around the protein.

The walls representing the membrane (*φ_5_*) follow the structure of the protein, allowing degrees of freedom inside the cavity. This end is achieved by building a set of points from the level-set function (Eq. 4). This surface, *φ_5_*, approximately follows the initial shape of the interface between protein and membrane (Fig. 1a). Choosing a relatively small value of *r_p_* around each atom, i.e. less than 1.5 Å, the whole protein may be overly constrained. A large value of *r_p_*, i.e. larger than 3 Å, allows water molecules to enter the region compressed between the wall and the protein. This may cause a distortion of the global fold of the protein. By testing different values in the range from 1.5 to 3.5 Å, we have found that the optimum value of r_p_ is 2.0 Å.

To avoid discontinuities, a larger radius (3*r_p_*) is first used, and then *φ_5_* is shifted back by 2*r_p_* (Fig. S1B).

The polar-aromatic residues (Tyr and Trp) have a specific affinity for a region near the lipid carbonyl in the membrane [3]. The presence of these residues has been shown to anchor the membrane protein in a lipid bilayer [3]. This effect has been taken into account by including not only the C*α* atoms but also all atoms of Tyr and Trp residues in the set of initial positions c*_j_* in Eq. 4, and by setting *r_p_*=1.4 Å for the side chain atoms of these residues. Hence the location of potential well, defined by *r_p_,* is closer to the wall, and the derived force is strengthened as well.

**References**:

1. Neri M, Anselmi C, Cascella M, Maritan A, Carloni P (2005) Coarse-Grained Model of Proteins Incorporating Atomistic Detail of the Active Site. Phys Rev Lett 95: 218102.

2. Neri M, Baaden M, Carnevale V, Anselmi C, Maritan A, et al. (2008) Microseconds dynamics simulations of the outer-membrane protease T. Biophys J 94: 71–78.

3. Killian JA, Heijne von G (2000) How proteins adapt to a membrane-water interface. Trends Biochem Sci 25: 429–434.
